# The Effects of Insect Infestation on Stored Agricultural Products and the Quality of Food

**DOI:** 10.3390/foods12102046

**Published:** 2023-05-18

**Authors:** Ioannis G. Stathas, Anastasios C. Sakellaridis, Marina Papadelli, John Kapolos, Konstantinos Papadimitriou, George J. Stathas

**Affiliations:** 1 Department of Food Science and Technology, School of Agriculture and Food, University of the Peloponnese, 24100 Kalamata, Greece; i.stathas@go.uop.gr (I.G.S.); a.sakellaridis@go.uop.gr (A.C.S.); m.papadelli@uop.gr (M.P.); i.kapolos@uop.gr (J.K.); 2 Laboratory of Food Quality Control and Hygiene, Department of Food Science and Human Nutrition, Agricultural University of Athens, Iera Odos 75, 11855 Athens, Greece; kpapadimitriou@aua.gr; 3 Department of Agriculture, School of Agriculture and Food, University of the Peloponnese, 24100 Kalamata, Greece

**Keywords:** carbohydrates, cereal, Coleoptera, legumes, Lepidoptera, lipids, proteins

## Abstract

In this review article, we focus on the effects of insect pests on the quality of stored cereals and legume grains. The changes in the amino-acid content, the quality of proteins, carbohydrates, and lipids, and the technological characteristics of the raw materials when infested by specific insects are presented. The differences reported concerning the rate and kind of infestation effects are related to the trophic habits of the infesting insect species, the variation of the component distribution in the different species of grains, and the length of the storage period. For example, wheat germ and brans feeders such as *Trogoderma granarium* may cause a higher reduction in proteins than endosperm feeders such as *Rhyzopertha dominica*, since the germ and brans contain higher concentrations of proteins. *Trogoderma granarium* may also cause higher reduction in lipids than *R. dominica* in wheat, maize and sorghum, in which most of the lipids exist in the germ. Furthermore, infestation with insects such as *Tribolium castaneum* may downgrade the overall quality of wheat flour, by increasing the moisture content, the number of insect fragments, the color change, the concentration of uric acid, the microbial growth, and the prevalence of aflatoxins. Whenever possible, the significance of the insect infestation and the concomitant compositional alterations on human health are presented. It should be highlighted that understanding the impact of insect infestation on stored agricultural products and the quality of food will be crucial for the required food security in the future.

## 1. Introduction

According to the Food and Agriculture Organization of the United Nations (FAO), 17% of the world food production today is destroyed during storage (10% by insects and 7% by mites, rodents, and diseases) [1]. The largest percentage (around 80%) of plant origin food is produced mainly from cereal grains (wheat, rice, maize, millet, sorghum, and barley) and legumes (beans, soya, and peanut), while 10% comes from other plants such as cassava, sweet potatoes, coconut, and bananas, and the remaining 10% comes from other plant species [2]. The insects that infest the aforementioned products during storage may have originated from the farm, as pests of the plants which are later transported to the storage area. They may also have infested the agricultural products during transport, processing or storage. The growth of insect populations in stored food is attributed to the favorable conditions that often prevail during storage and to the abundant nutrients present in the foods on which the insects grow [2].

Insect infestation of stored agricultural products causes significant quantitative damage [3,4,5,6]. However, in addition to the quantitative damage, the stored products are also degraded in quality, due to the presence of insects or the presence of their body parts in the products (wings, legs, molting, etc.), which are also undesirable in food. In addition, through their activity in food and the production of their metabolic products, insects contribute to the degradation of the quality of stored products by altering their chemical composition. The commercial and nutritional value of raw materials and foods is reduced, and consumers’ health problems may result [7,8].

The effect of insect infestation on stored food involves changes in the amino-acid and protein composition [9,10,11,12,13], in the available carbohydrates [13,14,15] and fats [10,16], and in the organoleptic characteristics [17,18]. The presence of insect populations in stored foods, in addition to the abovementioned impact, is often associated with microbial contamination. Fungi may produce mycotoxins such as aflatoxins, which are toxic to humans, affecting the quality and safety of foods [19,20,21]. Such cases of increased mycotoxin detection in stored foods due to insect activity have been reported for maize, almonds, peanuts, sweet potatoes, wheat, and rice [8,22].

In the present study, available data from the literature are reviewed, focusing on how insect infestation of stored cereal grains and legumes affects their composition, as well as the quality of certain food products.

## 2. Changes in Proteins Due to Insect Infestation

Cereals and legumes are a key source of carbohydrates, B vitamins, and certain trace elements, while covering a large percentage of nutritional requirements in protein. The protein content in cereal grains and in the marketable legumes fluctuates in the ranges 19–30% [23] and 18–43.1%, respectively [24]. For this reason, the effects of entomological infestations on their composition during storage constitute the subject of many studies in the literature.

The effects of the infestation by *Trogoderma granarium* (Everts) (Coleoptera: Dermestidae) and *Rhyzopertha dominica* (Fabricius) (Coleoptera: Bostrichidae) on the protein composition of cereal grains have been studied. Jood et al. examined the effects of a mixed population infestation of the above insects in a ratio of 50:50 on wheat, maize, and sorghum, where a reduction in the content of essential amino acids was recorded [9]. The maximum decrease was observed in methionine (38.9%) in wheat, in isoleucine (30.8%) in maize, and in lysine (32.9%) in sorghum. In this study, the chemical score index was determined, which expresses the protein quality as a function of the composition of its essential amino acids, defined as a percentage: (essential amino acids in protein/essential amino acids in egg protein) × 100. It was reported that, in the abovementioned cereals infested by *T. granarium* and *R. dominica*, the chemical score decreased significantly (*p* < 0.05) in all essential amino acids from 5.1% (in tryptophan) to 39% (in methionine). Regarding nonessential amino acids, an adverse effect on their content was also observed. The greatest reductions were observed in alanine and tyrosine in wheat, in histidine and cysteine in maize, and in serine and arginine in sorghum [9].

In another study, the effects of the infestation by *Prostephanus truncatus* (Horn) (Coleoptera: Bostrichidae), *Sitophilus zeamais* (Mots) (Coleoptera: Curculionidae), and *Sitotroga cerealella* (Olivier) (Lepidoptera: Gelechiidae) on essential and nonessential amino acids of the infested stored maize proteins were examined, and the authors recorded increased (+) and decreased (−) values [25]. In the case of *P. truncatus* infestation, the highest fluctuations of the essential amino acids between infested and uninfested products were recorded in phenylalanine (−19.0%) and in tryptophan (+36.8%) content. In the case of *S. zeamais* infestation, the corresponding highest fluctuations were recorded in glutamic acid (−49.5%) and tryptophan (+43.4%); in the case of *S. cerealella* infestation, they were recorded in cysteine (−24.6%) and tryptophan (+20.8%) [25].

**Figure 1:**
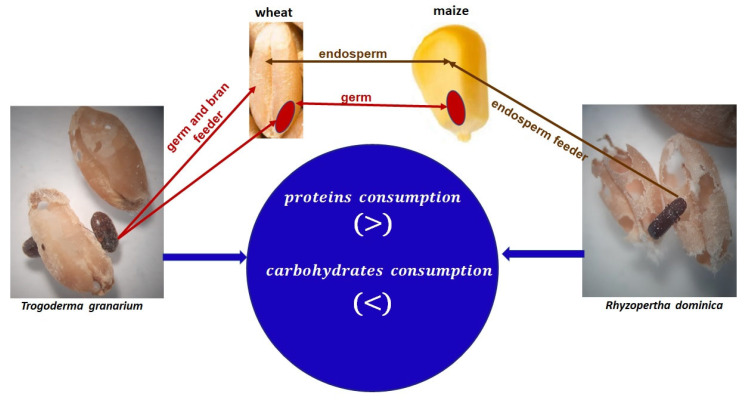
The potential influence of *Trogoderma granarium* and *Rhyzopertha dominica* pests on the proteins and carbohydrates in cereal grains.

To study the effects of insect infestation on the protein content of stored wheat, maize, and sorghum, the method of in vitro digestibility of proteins was used by Jood and Kapoor [26]. The cereals were infested by *T. granarium* and *R. dominica,* separately and in a mixed population, under infestation levels of 25%, 50%, and 75%. The protein digestibility of uninfested wheat was 72.9%, while that of wheat infested by *T. granarium* at 25%, 50% and 75% was reduced to 65.2%, 56.6%, and 45.5%, respectively. Similarly, the above levels of infestation by *R. dominica* led to a reduction of the protein digestibility to 68.0%, 61.3%, and 50.5%, respectively. The infestation by a mixed population of both species caused a decrease in the protein digestibility to 67.4%, 58.2%, and 47.3%, respectively. In the case of maize, the protein digestibility in uninfested grains was 71%, while that in grains infested by *T. granarium* at 25%, 50%, and 75% was reduced to 59.1%, 53%, and 41.1%, respectively. Similarly, the above levels of infestation by *R. dominica* led to a decrease in the protein digestibility to 64%, 60%, and 50.4%, respectively. The infestation by a mixed population of both species caused a decrease to 62.3%, 58.2%, and 48.1%, respectively. In the sorghum, *R. dominica* was the species that further reduced the protein digestibility from 64.5% of the uninfested sorghum to 55.6%, 48.0%, and 37.0%, compared to the reduction caused by *T. granarium* infestation (58.3%, 51.0%, and 43.3%) and the mixed population (61.2%, 57.8%, and 42.1%) at infestation levels of 25%, 50%, and 75%, respectively. The above differences were attributed to the different distribution of proteins and starch in the two cereal grains, and to the feeding preferences of each insect species. *T. granarium* mainly feeds on the germ and brans, while *R. dominica* feeds on the endosperm. Additionally, the percentage of proteins is differently distributed in each cereal species and in each part of the grain. In wheat, a higher concentration of protein exists in the germ (26%) and brans (48%); this is also seen in maize (34.4% and 13.2%, respectively), in contrast to sorghum, in which protein mainly exists in the endosperm (80.9%). Accordingly, *T. granarium* caused a higher reduction in protein digestibility in wheat, whereas *R. dominica* caused a higher reduction in sorghum [26] (Figure 1).

Infestations of stored wheat, maize, and sorghum grains by *T. granarium* and *R. dominica* caused a significant reduction in protein nitrogen and true protein content, but an increase in the total nitrogen, total protein, non-protein nitrogen, and uric acid content, due to the insect fragments and the insects’ metabolic products [27]. Additionally, *R. dominica* being mainly an endosperm feeder, caused more losses of available carbohydrates as compared to germ feeder *T. granarium* [14].

The fluctuation and the protein quality in cereal grains can be evaluated biologically by the definition of the following indices: food efficiency ratio (FER), protein efficiency ratio (PER), biological value (BV), net protein utilization (NPU), net protein retention (ΝΡR), and protein retention efficiency (ΡRΕ) [28]. These indices were studied in experiments measuring the growth of rats fed by wheat grains and showed a negative correlation with the infestation level of the grains by *T. granarium* and *R. dominica* [28]. A similar study using rats was conducted to evaluate the nutritive value of infested sorghum grains by *Tribolium castaneum* (Herbst) (Coleoptera: Tenebrionidae). The results showed that over 30% infestation of seeds caused a significant degradation of proteins [10].

Ahmedani et al., studying the effects of *T. granarium* infestation on the wheat *Triticum aestivum*, found an increase in the proportion of total proteins, after 6 months of storage [29]. Mendes et al. studied the quality of proteins in bean grains infested by *Acanthoscelides obtectus* (Say) (Coleoptera: Bruchinae) and corn and wheat grains infested by *S. zeamais*. The authors found that the indices PER and ΝΡR decreased in the infested beans but not in the infested grains of corn and wheat [30].

Nta et al. revealed that the infestation by *A. obtectus* of *Phaseolus vulgaris* and *Phaseolus acutifolius* caused a reduction in protein levels in beans [31]. Gabarty et al., studying the effects of infestation of wheat flour by *Corcyra cephalonica* (Stainton) (Lepidoptera: Pyralidae), *Ephestia kuehniella* (Zeller) (Lepidoptera: Pyralidae), and *Tribolium confusum* (du Val) (Coleoptera: Tenebrionidae), recorded a significant increase in the total protein contents of the flour [5]. As mentioned above, in some studies, the cereals infested by insects exhibited an increased total protein content and a reduced true protein content. The reduction in true protein (or beneficial proteins) could be attributed to the feeding activity of the insects. The increase in total protein percentage could be related to the production of nonbeneficial (rather harmful) proteins originating from the bodies of the insects and their fragments such as exuviates, wings, and legs that are found in the samples of the grains. Alternatively, total protein content may be increased due to by-products of the insects’ metabolism. Furthermore, the increase in protein percentage could be the result of the significant reduction in carbohydrate percentages [29].

## 3. Changes in Carbohydrates Due to Insect Infestation

The available carbohydrates (soluble sugar, reducing sugar, nonreducing sugar, and starch) of wheat, maize, and sorghum grains, affected by storage and infestation by *T. granarium* and *R. dominica*, were studied by Jood et al. [14]. Each of the insect species was employed separately or in mixed populations, under different infestation levels of the grains at 25%, 50%, and 75%. It was found that *R. dominica* caused a significant (*p* < 0.05) reduction in available carbohydrates at 50% and 75% infestation levels, whereas *T. granarium* caused a similar effect at 75%. The mixture of both insect species caused intermediate losses. The composition of the carbohydrates was affected by the duration of the storage period. When the storage period was longer than 4 months, a significant increase in sugars and a significant decrease in starch content were recorded.

Nta et al. showed that the infestation by *A. obtectus* of *P. vulgaris* and *P. acutifolius* beans caused a reduction in carbohydrates [31]. The percentage of reduction fluctuated between 10.2% and 30.5%, depending on the infestation level and the bean species. Gabarty et al. also found that total carbohydrates (monosaccharaides and disaccharides) were significantly reduced in wheat flour infested by *C. cephalonica*, *E. kuehniella*, and *T. confusum* [5]. Similarly, it was reported that the increase in infestation level of *S. zeamais, P. truncatus*, and *Callosobruchus maculatus* (Fabricius) (Coleoptera: Bruchidae) in stored maize and cowpea promoted a decrease in carbohydrates and an increase in the percentage of proteins due to the consumption of carbohydrates by the insects [13].

A parameter of carbohydrates related to insect infestation is the glycemic index (GI), which was introduced for the classification of carbohydrates in carbohydrate-rich foods. It is a value rated from 0 to 100 that ranks the foods according to the rate they supply the blood with glucose. It basically indicates the rate of food digestibility. The rate of blood supply with glucose and the duration of increased glucose in blood may cause hormonal and metabolic changes that may affect parameters related to health. The foods with a high GI are considered harmful for health. Inversely, a low GI of foods is often beneficial for certain chronic diseases. For this reason, it was proposed that GI data for foods can be used to make priorities for food selection within food groups [7]. Kumar et al. studied the influence of *S. cerealella* infestation on the GI of five different genotypes of stored paddy rice [15]. In this study, the authors found that the insect infestation caused a reduction in the values of amylose content (AC), total carbohydrate (TC), and resistant starch (RS), as well as an increase in the values of glycemic load (GL). The effects of infestation varied across the different genotypes of rice, due to the different composition of grains of each genotype. On head rice (milled rice with the whole kernel or greater than 75% intact length), a positive relation between the infestation level and increase in GI was recorded, while a negative relation was observed between the infestation level and resistant starch content [15].

## 4. Changes in Lipids Due to Insect Infestation

Cereal grains consist of about 10% lipids [23]. Lipids are a rich source of energy, supplying 9 kcal/g. They are fatty acids and glycerol, nonpolar molecules that do not dissolve well in water [32]. Polyunsaturated fatty acids are beneficial for nutrition but can be oxidized during the period of the storage, leading to off-flavors [33]. The effects of insect infestation and storage duration on lipids of cereal grains were studied by Jood et al. [16]. In this study, wheat, maize, and sorghum grains were infested by *T. granarium* and *R. dominica* at different infestation levels (25%, 50%, and 75%) separately or in mixed populations. A significant reduction in the content of total lipids, phospholipids, galactolipids, and polar and nonpolar lipids was observed at 50% and 75% infestation levels (*p* < 0.05), suggesting that lipid levels negatively correlated with the infestation level. Most lipids in all three cereal grains exist in the germ, while few exist in the bran and endosperm. The distribution of lipids is higher in the germ than the endosperm and bran for wheat, maize and sorghum, respectively [16]. This is the reason that the mainly germ feeder *T. granarium* caused a higher decrease in lipids than the endosperm feeder *R. dominica* in the three cereals. In addition to the infestation level, the effects of the duration of storage period of the cereals on the lipid concentration were examined. For all storage periods (1, 2, and 4 months), no significant changes to the content of total lipids and other classes of lipids were observed [16].

## 5. Qualitative and Technological Characteristics

Insect infestations, in addition to the above alterations on the composition of stored foods, including constituents such as proteins, carbohydrates and lipids, cause alterations in their metabolic products, such as purines, quinones, and uric acid [10,17,27,34], which affect the organoleptic characteristics and the suitability of the products for processing and consumption. Insect infestations of cereals and legumes also affect other components of the grains. Pant and Susheeda recorded a significant reduction in total fat, mineral matters, and vitamins thiamin and riboflavin in highly infested (30%) sorghum grains by *Tribolium castaneum* (Herbst) (Coleoptera: Tenebrionidae) [10]. In legumes (*P. vulgaris* and *P. acutifolius*), the infestation by *A. obtectus* caused a reduction in minerals Na, Mg, Fe, and Co and an increase in K and Zn [31]. Furthermore, *C. maculatus* pest on cowpea seeds caused changes in the composition of the essential elements, reducing the concentration of Al and Fe and increasing that of Mn [35]. Uric acid is a degradation product of purines, which is an indicator of insect infestation and the state of stored grains [36]. Wehling et al., using liquid chromatography, studied the uric acid level of wheat infested by *Sitophilus granarius* (L.) (Coleoptera: Curculionidae), *Sitophilus oryzae* (L.) (Coleoptera: Curculionidae), and *R. dominica*, and found that a positive correlation exists between the infestation level and uric acid content of grains [37]. The baking and taste quality of bread prepared from wheat flour infested by *Oryzaephilus surinamensis* (L.) (Coleoptera: Silvanidae), *Tenebrio molitor* L. (Coleoptera: Tenebrionidae), *T. castaneum*, and *T. confusum* were studied [34]. For bread prepared with flour infested by *O. surinamensis* and *T. molitor*, minor changes were recorded in its physical characteristics, while a ‘chemo-phenolic’ taste and odor were observed. The infestation by *T. castaneum* and *T. confusum* resulted in the appearance of a progressive darkening of the crumb, a reduction in slice size, and a distinct offensive taste and smell, which were created during the infestation period. The above characteristics could be attributed to quinone secretions by the *Tribolium* species in the flour, which may be an important factor downgrading its baking qualities [34]. Another study was also performed to examine the effects of *R. dominica* and *T. granarium* infestation on wheat, maize, and sorghum grains on the organoleptic characteristics and quality of bread (type ‘chapatis’) prepared with the corresponding flours [17]. The differences in the color, taste, texture, aroma, and appearance between the products prepared with infested and uninfested grain flours were recorded. It was found that only the taste of bread prepared with the flour from the infested cereals under infestation levels of 50% and 75% was significantly affected compared to the one prepared with the flour from the uninfested grains (*p* < 0.05). A bitter taste also resulted in poor overall acceptability of chapatis prepared with flour infested at the 50% and 75% levels. The change in taste could be attributed to the change in the composition of the infested cereals. At the insect infestation levels of 50% and 75% in the three abovementioned cereals, increased quantities of uric acid and antinutrients such as polyphenol and phytic acid were detected. Reductions in protein nitrogen, in starch and protein digestibility [26], in minerals (Ca, P, Zn, Fe, Cu, and Mn) [38], and in available carbohydrates [14] were also reported.

Özkaya et al. [39] studied the technological properties of soft and hard bread wheat flour infested by *T. confusum* and *R. dominica*. *T. confusum*, feeding on the germ of grains without damaging the endosperm, produced little debris, while *R. dominica*, consuming both the endosperm and the germ, produced high amounts of debris. Bread prepared with infested flour had a reduced loaf volume and a significant change in odor and taste after 2 months of infestation period [39].

The rheological properties of flour from wheat grains infested with *R. dominica* were reported by Perišić et al. [40]. The quality parameters examined were the wet gluten and gluten index of two varieties of wheat. The infestation caused a lower gluten index content, greater weakening of the gluten network upon mixing and heating, and lower starch gelatinization viscosity. Although the changes in rheological properties were mainly dependent on the infestation level, the type of varieties also influenced the degree of the recorded changes [40]. Another study [41] described similar effects of *R. dominica* pest on wheat from triticale and rye, on wet gluten content and structure, and on starch damage. In this study, the use of diatomaceous earth controlled the population of the insect, limited the above unpropitious changes, and downgraded the rheological properties of the examined cereals. *Sitophilus granarius* is another species affecting the technological properties of wheat. Keskin and Ozkaya evaluated the effects of *S. granarius* infestation of wheat on the physical, chemical, and physicochemical properties of the flour obtained from the varieties Ceyhan-99 (hard wheat) and Eser (soft wheat) [42]. A higher insect population was developed in the soft wheat variety. The increase in *S. granarius* population caused a decrease in the kernel and test weight of grains, determined using an Ohaus test weight apparatus, as well as in the fat content, the gluten content, the gluten index, and the sedimentation value; in contrast, it increased the ash content, protein content, and acidity value. The increases in protein and ash content might have been the result of high bran contamination. This occurs because *S. granarius* mainly feeds on the endosperm and germ layers, causing a reduced ratio of endosperm to bran. Additionally, the highest concentration of proteins in wheat grains exists in the bran. Moreover, the presence of insect body parts in the flour and the metabolic secretions of the insect may be additional reasons behind the increases in protein and ash [42]. A further study suggested that *S. granarius* infestation of meal flour caused an increase in the acidity of the flour and its protein solubility (PS) value [43]. In rice grains infested by insects, volatile organic compounds (VOCs) detected using a chemometric method were found to be released. Additionally, a correlation between the rice infestation by *S. oryzae* and *R. dominica* and the population of the fungi of the genus *Aspergillus* and *Penicillium* was documented [8].

The possible synergistic interaction among the insect infestation of wheat flour by *C. cephalonica*, *E. kuehniella*, and *T. confusum,* the fungal population (genera *Aspergillus*, *Penicillium*, *Cladosporium*, *Eurotium* and *Emericella*, and mycotoxin presence, was studied by Gabarty et al. [5]. The authors found that infestation of flour by the above species greatly increased total mold populations. This could be attributed to the production of metabolic heat and water by insects in stored flour. Additionally, the insects carry fungal spores on their body, increasing fungal counts. The mycotoxins detected in wheat flour infested by the insects mentioned above, after 2 months of storage, were aflatoxins B1 and B2 and ochratoxin A. Aflatoxins B1 and B2 were weakly detected in the uninfested flour and in that infested by *E. kuehniella*. A moderate detection was recorded in flour infested by the three insect species together, while a strong detection was recorded in flour infested by *C. cephalonica* and *T. confusum*. No detection of ochratoxin A was recorded in uninfested flour and that infested by *C. cephalonica*. A weak ochratoxin A detection was recorded in the case of infestation by *E. kuehniella*, a moderate one was recorded for the infestation by a mix of the three insect species, and a strong detection was recorded in the flour infested by *T. confusum* [5]. Mycotoxins are very toxic for humans, potentially causing teratogenesis, mutagenesis, carcinogenesis, deformity, mutation, allergic reactions, death, etc. [44,45,46]. The presence of additional metabolites of insects in infested cereals were determined by Tanaka et al. [47].In particular, the infestation of brown rice by *S. zeamais* and *Plodia interpunctella* (Hübner) (Lepidoptera: Pyralidae) caused the production of one or more, of the volatile compounds: prenol, isoprenol, dimethyl disulfide, and dimethyl trisulfide.Negi et al. presented the effects of *T. castaneum* infestation on the quality of wheat flour, concerning moisture content, insect fragment existence, color change, uric acid concentration, microbial growth, and aflatoxin presence [18]. The moisture content of the flour increased with the increase in insect population, due to metabolic activity and aerobic respiration. The increase in moisture resulted in the growth of bacteria and fungi. Microbial spores and fungi were spread by the insects and their fragments. *Aspergillus*, *Rhizopus*, *Penicillium*, and *Saccharomyces* were found in flours with the higher moisture levels and in flours containing insect fragments.

Magagnoli et al. demonstrated that the infestation of maize by *Ostrinia nubilalis* (Hübner) (Lepidoptera: Crambidae) can cause contamination with aflatoxigenic fungi, such as *Aspergillus flavus*, the major producer of aflatoxin B1 [48]. It was reported that, when maize grains were infested by *O. nubilalis* under favorable climatic conditions, high mycotoxin concentrations were usually found in grains [48]. The infestation of maize and cowpea by *S. zeamais*, *P. truncatus*, and *C. maculatus*, in addition to the abovementioned decrease in carbohydrates and increase in proteins, demonstrated a positive correlation between the infestation and moisture percentage. The quantity of carotenoids was independent of insect infestation, but related to the period of storage, being reduced after a storage period of 4 months [13].

In addition to stored cereals, corresponding damages can be noted in the majority of infested stored products. Infestation by *Stegobium paniceum* (L.) (Coleoptera: Anobiidae) and *Lasioderma serricorne* (Fabricius) (Coleoptera: Anobiidae) of coriander powder, sambar powder, and turmeric rhizomes was studied [49]. After 3 and 6 months of infestation, there was a significant reduction in the protein, fat, and ash contents of all products studied, whereas the moisture content increased in coriander and sambar powders. The uric acid levels exceeded the permissible limit of 100 mg/100 kg after 3 or 6 months of infestation in all cases [49].

## 6. Human Health and Insect Pests

Many studies in the literature revealed the emergence of human diseases that are related to insect existence in foods. The harmful effects of insect infestations on human health are due to the direct consumption of insects or their parts (wings, legs, and molting) that constitute allergenic factors, the insects’ metabolic products accumulated in the infested foods, contamination with pathogens, and the aflatoxins and other mycotoxins produced by fungi. These factors should also be taken under consideration in cases of edible insects used as human or animal food [50].

In an investigation studying bakers’ asthma in France, it was found that, among other factors, insects of the genera *Sitophilus* and *Ephestia* and the species *T. molitor* stimulated asthma [51]. In another study, it was found that among the arthropods infesting the stored grains, the most important sources of allergens were mites and the insect species *T. castaneum*, *Cryptolestes ferrugineus* (Stephens) (Coleoptera: Laemophloeidae), *O. surinamensis*, *S. oryzae*, and *R. dominica* [52].

Molds have also been confirmed as potential agents that cause allergies. However, it is likely that the major problem resulting from the presence of molds in food is due to the toxic effects of mycotoxins, rather than their allergenic effects. Thus, the adoption of good management and processing techniques can help to minimize the risk [53]. The effect of insect pests on the GI mentioned above may also be considered to be harmful for human health [7,15]. Lastly, insect proteins can lead to allergies [54].

## 7. Future Perspectives and Concluding Remarks

The presence of pests in agricultural products and foods is strictly regulated in all parts of the world to protect human health and to prohibit the spread of the pest to foreign ecosystems during trade. The consequences of insect infestations on the composition of the individual constituents of stored grains and legumes were reviewed herein. In addition to the degradation of the quantity and quality of the products, pests are also related to adverse effects on the health of the consumers. The influence of the pests on the contents of stored products varies depending on the insect species, the kind of stored agricultural products, the storage period, and the infestation level. Furthermore, the variation of infestation effects is dependent on the different feeding habits of the insects and on the different distribution of the contents in the grains of each cereal species. The differences in effect between the infestation by *T. granarium* and *R. dominica* of wheat, maize, and sorghum on proteins, carbohydrates, and lipids could be attributed to the different feeding preferences of the species, in combination with the different distribution of these substances in the grains of cereals. Similarly, differences in insect pest effects were presented in legumes. As also discussed above, certain insects have been found to alter the content of minerals. Among the major effects of insect pests on stored foods are the downgrade in baking and the flavor quality of bread prepared from wheat flour infested by *O. surinamensis*, *T. molitor T. castaneum*, and *T. confusum* [34]. In addition to the negative effects on the technological characteristics of the infested foods, the harmful effects on human health should be taken into account. It was recommended that *S. cerealella*-infested rice grains must be assessed for grain quality and composition before consumption to reduce the health risk [15]. Other effects that could be harmful for human health in wheat flour infested by *T. castaneum* are the increase in moisture content, the existence of insect fragments, uric acid concentration, microbial growth, and the presence of aflatoxins [18].

The studies mentioned above examined the consequences of insects on the quality of stored agricultural products, following infestation of cereals and legumes, as they provide the largest percentage (80%) of plant origin food. Most of the referred insect species belong to the order Coleoptera (15 species), which is the largest order of the class Insecta, while the rest belong to the order Lepidoptera (four species). The range of the multilateral effects of insect infestation on stored food and its importance for food quality and human health indicate the necessity of adopting effective methods for its control.

It should be noted that changes in plant physiology and metabolism can result from insect infestations even before harvest. These changes are attributed to an interaction between plants and insect pests, through the creation of a dynamic defense mechanism in plants against the insect pests. Vo et al. [55] applied proteomics and metabolomics studies on rice after insect infestations. Changes in amino-acid transport and metabolism, as well as in photosynthesis, are caused by sucking insects such as *Nilaparvata lugens* (Stål) (Hemiptera: Delphacidae) and chewing insects such as *Cnaphalocrocis medinalis* (Guenée) (Lepidoptera: Pyralidae). Similarly, chemical analytical techniques were employed to describe metabolic changes in maize plants, resulting from the triggering of a chemical defense mechanisms of this cereal against insect pests [56]. The chemical responses of maize plant against the infestation by the species *Diabrotica virgifera virgifera* (LeConte) (Coleoptera: Chrysomelidae), *Spodoptera littoralis* (Boisduval) (Lepidoptera: Noctuidae), and *Spodoptera frugiperda* (Smith) (Lepidoptera: Noctuidae) were studied. Marti et al., using ultrahigh-pressure liquid chromatography/time-of-flight mass spectrometry (UHPLC–TOF-MS), determined the changes in the metabolite profile at the local and systemic levels in maize plants infested by *S. littoralis*, giving 32 differentially regulated compounds [57].

The use of metabolomics and other omics methods can contribute to obtain further information for monitoring and controlling insect pests in stored cereal and legume products. Many of these insect species infest the plants during the cultivation period, when the metabolite profile can be affected. Furthermore, infestation of a plant by an insect species can induce the production of defensive metabolites in the plant against another insect species. For instance, the underground pest *D. virgifera virgifera* stimulates a plant defense mechanism on maize leaves against the aboveground pest *S. littoralis* [56]. Further studies related to metabolomics and other omics methods on stored cereals and legumes can give very important information concerning the interpretation or the detection of insect pests on stored foods. In a world that is confronted with the critical challenge of food security, minimizing the losses of agricultural products and food from insect infestations will be monumental. Understanding the infestation process in depth may provide new ways to combat it.
